# Surface Hydrophobic Modification of Microcrystalline Cellulose by Poly(methylhydro)siloxane Using Response Surface Methodology

**DOI:** 10.3390/polym10121335

**Published:** 2018-12-03

**Authors:** Yulin Xie, Siquan Cai, Zhen Hou, Weihua Li, Yan Wang, Xinxiang Zhang, Wenbin Yang

**Affiliations:** College of Material Engineering, Fujian Agriculture and Forestry University, Fuzhou 350002, China; Xieyulin1217@163.com (Y.X.); caisiquancsq@163.com (S.C.); 13965835371@163.com (Z.H.); 13395939925@163.com (W.L.); wangyan001010@163.com (Y.W.); xxzhang0106@163.com (X.Z.)

**Keywords:** response surface methodology, poly(methylhydro)siloxane (PMHS), microcrystalline cellulose (MCC), hydrophobic modification, water contact angle

## Abstract

Poly(methylhydro)siloxane (PMHS) and n-hexane were used as modifiers and solvents, respectively, to prepare surface modification of microcrystalline cellulose (MCC). The response surface methodology was used to optimize the effects of reaction conditions on hydrophobicity of MCC. The optimal reaction conditions were determined as follows: the concentration of PMHS was 0.0275% (the mass ratio of PMHS to MCC), the reaction time was 20 min, and the drying temperature was 70 °C. Under the optimum reaction conditions, the water contact angle of modified MCC was 141.5°. It is feasible to optimize and select the reaction conditions of modified MCC by Design-Expert, and the predicted value of the mathematical model is in good agreement with the experimental value. Surface chemical characteristics were investigated using X-ray photoelectron spectroscopy (XPS). These analyses confirmed that the PMHS chains were attached to MCC. Due to the introduction of a large amount of methyl groups, the reaction between MCC and PMHS leads to an improvement in its hydrophobicity.

## 1. Introduction

Microcrystalline cellulose (MCC) has advantages of wide source, exceptional mechanical properties, high temperature resistance and improved liquid stability. These give it great potential for the reinforcement of composite materials [1,2]. MCC is quite hydrophilic while most polymer matrices are hydrophobic. Due to the inherent incompatibility between the hydrophilic MCC and the hydrophobic polymer matrix, the aggregation of MCC in the polymer matrix is a major challenge in the fabrication of MCC reinforced polymer composites. The chemical modification of MCC is an effective method to improve the interfacial compatibility of subsequent matrix polymers in composite preparation [3,4]. For example, MCC reinforced polypropylene (PP) composites were developed by using maleic anhydride (MA) [5] and ma-graft-PP (MAPP) [6,7] as coupling agents, respectively. Since the hydroxyl group of MCC reacts with the isocyanate group of the polyurethane resin system, MCC is used as a reaction enhancer to prepare MCC reinforced polyurethane (PU) composites [8,9]. MCC and methyl methacrylate (MMA) have been used to develop new cellulose-based copolymers in aqueous media that exhibit higher thermal stability and better compatibility with natural rubber [10,11]. In summary, numerous methods of reinforcing different matrix polymers by MCC have been extensively studied. However, the modification method in most of the works was still cumbersome, the reaction cycle was long and hydrophobic modification also did not obvious.

This paper presented a method for hydrophobic modification of MCC surface by PMHS. This method possesses the advantages of rapid reaction at room temperature and low cost of modifier. PMHS is a polydimethylsiloxane with a large amount of reactive Si-H bond and hydrophobic-CH_3_ in the main chain. The molecular formula is (CH_3_)_3_SiO(CH_3_HSiO)_m_((CH_3_)_2_SiO)_n_Si(CH_3_)_3_, according to the values of m and n, it is divided into PMHS with different hydrogen content. The Si-H bonds on the PMHS chain and the –OH groups on the surface of MCC will undergo the dehydrogenation reaction in the presence of catalyst. The hydrophobic PMHS chain is introduced into the MCC and modified MCC was successfully fabricated. After modification, the hydrophobicity was significantly improved, which was beneficial to solve the problem of interface compatibility between MCC and matrix polymer.

Computer Design-Expert software was used to design the experiments. The response surface method is a statistical method to solve the multivariable problem by establishing the function between the factors and response values of the multiple quadratic regression equation and obtaining the optimal process parameters [12,13] through the analysis of the regression equation. In this method, the response value under multiple variables can be obtained through the regression fitting equation, response surface and corresponding contour plots [14], and the experimental conditions can be optimized. In this study, Design-Expert Design method was used to optimize the reaction conditions of modified microcrystalline cellulose.

## 2. Experimental Section

### 2.1. Materials

Microcrystalline cellulose (MCC) was purchased from Tianli Pharmaceutical Auxiliary Materials Co., Ltd., Qufu, China. Poly(methylhydro)siloxane (PMHS) (with reactive hydrogen content of 1.5%) and Karstredt catalyst (Pt content: 3000 pm) were supplied by Zhonglan Chenguang Chemical Research and Design Co., Ltd., Chengdu, China. *N*-hexane was obtained from Tianjin Zhiyuan Chemical Reagent Co., Ltd., Tianjin, China. All chemicals were used as received without further purification.

### 2.2. Surface Modification of MCC

In a 100-mL beaker equipped with a magnetic stirring bar, 0.5 g MCC, 50 g n-hexane, a certain mass fraction of PMHS and 1 to 2 drops of catalyst were added. The mixture was then stirred at 25 °C for a period of time using a magnetic stirrer. After reaction, the sample was filtered through 0.22 μm PVDF (polyvinylidene fluoride) filters and washed three times with n-hexane. Finally, the modified MCC powder was obtained by drying in an oven at a certain temperature for three hours.

### 2.3. Water Contact Angle (WCA) Measurement

The WCA was measured using a sessile drop configuration at room temperature on the HARKE-SPCA-2 instrument (Harke, Beijing, China), equipped with a camera. A small piece of double-sided tape was applied to the slide, and the MCC powder was evenly spread over the double-sided tape surface and compacted with another clean glass slide. The water in all measurements was of high purity and 5 μL of water droplets per measurement. Each sample was measured three times and finally the data was recorded as an average.

### 2.4. Statistical Methods

In this experiment, the response surface method was used to optimize the optimal reaction conditions for modifying the hydrophobic properties of MCC. Three main factors influencing the hydrophobic properties of modified MCC are relative quantity of PMHS, reaction time and drying temperature. The experiment was designed according to the Design-Expert software. The predicted and actual values of the experiments were compared and the quadratic regression equation was fitted to optimize the reaction conditions.

### 2.5. X-ray Photoelectron Spectroscopy (XPS)

The XPS spectra were recorded on an ESCALAB 250 (Thermo Scientific Instruments Co., Ltd., Waltham, MA, USA) equipped with excitation source for the monochromatic source of aluminum and potassium, fixed through energy analyzer for 100 eV, 650 μm light spot size, the scanning range of 0~1400 eV.

## 3. Results and Discussion

### 3.1. Hydrophobic Analysis

**Figure 1:**
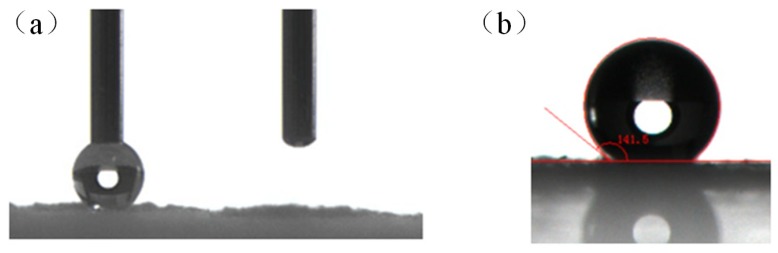
The WCA of unmodified MCC (**a**), the WCA of modified MCC (**b**).

To realize modified MCC with good hydrophobicity, the water contact angles (WCA) were investigated. As shown in Figure 1a, the water drop is fully absorbed as soon as it touches the unmodified MCC powder, indicating the MCC was very hydrophilic. This is because unmodified MCC contains an abundant number of –OH groups [15]. Figure 1b showed the water contact angle of the PMHS modified MCC. The optimum reaction conditions are optimized by the Design—expert software as follows: the concentration of PMHS was 0.0275%, the reaction time was 20 min, the drying temperature was 70 °C. After optimization, the water contact angle was tested to be 141°. This was probably because PMHS chains were introduced to the surface of MCC.

**Figure 2:**
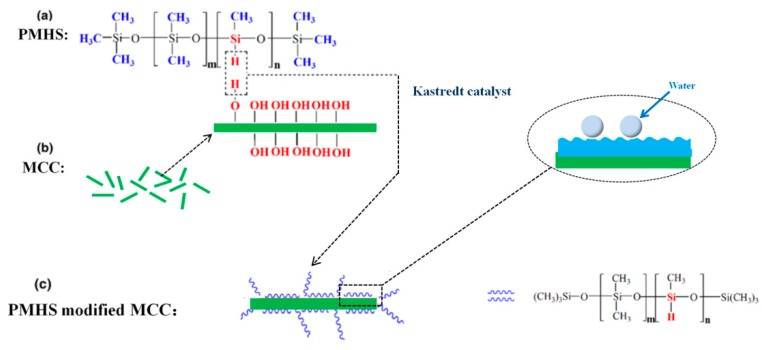
The schematic representation of PMHS (**a**), MCC (**b**), and PMHS modified MCC (**c**).

The surface modification of MCC by PMHS is based on the rapid reaction between the hydroxyl groups on the MCC surface and reactive –Si–H bonds of the PMHS chains. As shown in Figure 2a, the PMHS chain has several reactive –Si–H bonds and a large quantity of hydrophobic –CH_3_ groups. The Si–H bonds are highly reactive and can undergo a variety of chemical reactions, especially materials containing vinyl and hydroxyl groups. The presence of several –CH_3_ groups make the PMHS surface energy very low. As shown in Figure 2b, MCC is abundant in hydroxyl groups. Karstedt catalyst is the complex compound of platinum and 1,3-divinyl-1,1,3,3-tetramethyldisiloxane. It has strong catalytic activity for dehydrogenation between –Si–H bands of PMHS and hydroxyl groups of MCC at room temperature. The MCC sample was immersed in a modified solution containing PMHS, n-hexane and Karstedt catalyst and immediately produced a large number of bubbles. As shown in Figure 2c, it can be inferred that a large number of PMHS chains will be grafted onto the MCC surface in this simple method.

### 3.2. Experimental Design and Results

The design-expert software provides two different methods to display the level of each factor in the experimental design: (1) the actual levels of the factors (i.e., the actual values in the experiment) and (2) the coded factor levels (i.e., as −1 for low levels, +1 for high levels, and 0 for centrepoint). The actual and coded factor levels are shown in Table 1. The experimental design matrix is represented by the coding factor level generated by the design expert software, is shown in Table 2. The predicted value of WCA is obtained from the data report of the Design-expert software, which is calculated by the Design-expert software based on the regression equation.

### 3.3. Analysis of Regression

The experimental results of WCA were input into the software for regression analysis, and the most suitable model equation was obtained. The derived quadratic multiple regression equation describing the relationship between the WCA and the three variables is: WCA = 140.23 + 1.66 × A − 0.76 × B + 1.11 × C + 1.19 × AB + 1.95 × AC − 2.78 × A^2^ − 1.64 × B^2^ − 2.09 × C^2^ − 1.73 × A^2^C.

**Figure 3:**
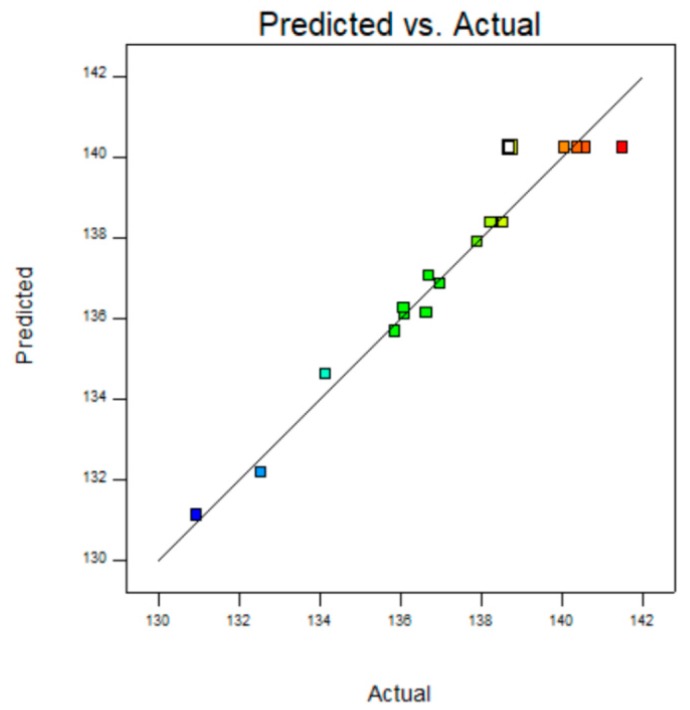
Predicted value and measured value of WCA.

The statistical significance was check by the F values, *p* value and the adequate precision ratio. The lower the *p* value, the higher the statistical significance of the variable; *p* value less than 0.05 is significant, while greater than 0.1 is not significant. Table 3 showed the experimental variance analysis, it could be seen that the model F value of 19.35 implied the model was significant and the *p* value of 0.0004 also implied that the model was significant [16,17]. The Lack of Fit *p*-value of 0.8269 implied that the Lack of Fit was not significant relative to the pure error. This equation is well fitted to the actual situation [18], and could reflect the relationship between concentration of PMHS, reaction time, drying temperature and hydrophobic performance of modified MCC. Figure 3 showed the comparison between experimental predicted value and experimental value. It could be seen from the figure that the test quantity was close to a straight line, indicating that the actual value was close to predicted value [19].

### 3.4. Variable Analysis

#### 3.4.1. Response Surface Methodology Analysis

The response surface and the corresponding contour plots were established and the main effect and interaction effect of the experimental variables and their contribution to the prediction of the research response are evaluated.

**Figure 4:**
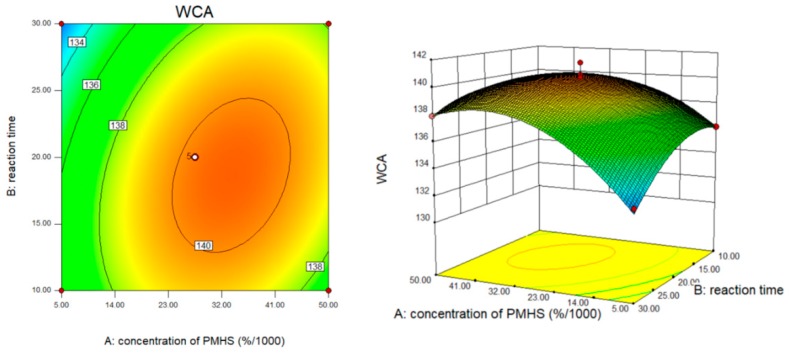
The effect of PMHS concentration and reaction time.

**Figure 5:**
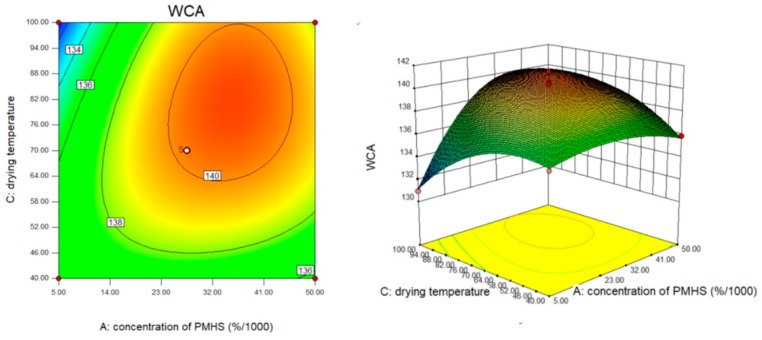
The effect of PMHS concentration and drying temperature.

Figure 4 showed the effects of PMHS concentration and reaction time on the WCA of modified MCC. At low PMHS concentration, the PMHS could not provide the sufficient Si–H bond to dehydrogenation reaction. A further increase in concentration of PMHS did not result in an obvious increase in WCA. This may be due to the entanglement between the chains of PMHS itself, which entraps the –Si–H bond, resulting in only a small amount of –Si–H bonds reacting with the hydroxyl groups of MCC. Under the condition that PMHS concentration remains unchanged, WCA of modified MCC increases with the increase of reaction time, especially within the reaction time range of 10–20 min, WCA gradually increases with the increase of PMHS concentration, from 0.005% to 0.05%. The contour diagram was elliptic, indicating that PMHS concentration and reaction time had significant interaction with WCA. The interaction between PMHS concentration and drying temperature was shown in Figure 5. The WCA of modified MCC increased with the drying temperature from 40 to 70 °C at some PMHS concentration. Excessive temperature led to the decrease of WCA, it might because of PMHS decomposition on MCC surface [20]. 

Furthermore, the optimal conditions to obtain the highest WCA of modified MCC were determined as follows: PMHS concentration 0.0275%, reaction time 20 min, and drying temperature 70 °C. Under the optimal conditions, the obtained WCA of modified MCC was 141.5°.

#### 3.4.2. Experimental Optimization Verification

**Figure 6:**
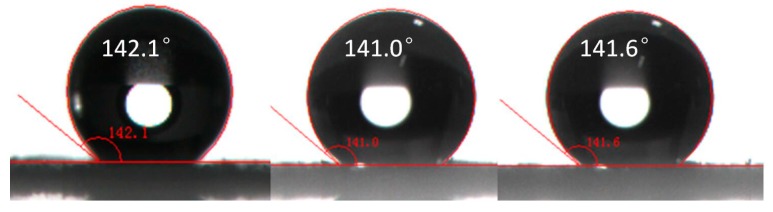
The WCA of verification experiment.

To verify the accuracy the predicted values of the response surface method and the regression equation. The experiment was repeated under the optimized experimental conditions. The WCA of samples was measured 3 times at different positions. As shown in Figure 6, under the optimum reaction conditions, water contact Angle was basically stable at around 141°. It indicated that the response surface method was effective for the optimization of MCC hydrophobicity, and had forecast guidance to the experiment.

### 3.5. XPS Analysis

**Figure 7:**
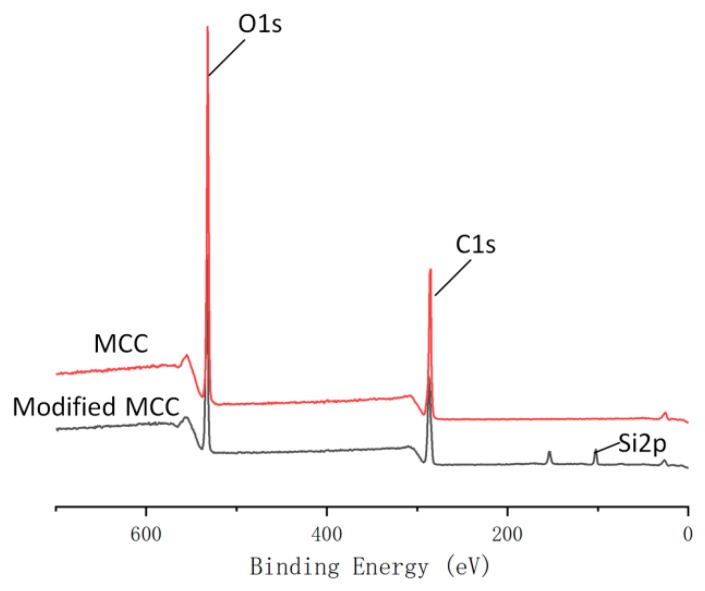
XPS spectra of unmodified MCC and modified MCC.

XPS was used for qualitative analysis of element composition. This spectral technique is a surface sensitive technique that can provide information about chemical changes on the surface. Figure 7 indicated the surveyed spectra of MCC and modified MCC. It could be seen that both MCC and modified MCC samples showed peaks of C1s and O1s at 285.17 and 531.17eV, respectively [21,22]. For modified MCC, there are two new Si2p and Si2s from PMHS, which emerged at 101.17 and 152.3 eV [23]. It indicated that PMHS successfully reacted with MCC.

**Figure 8:**
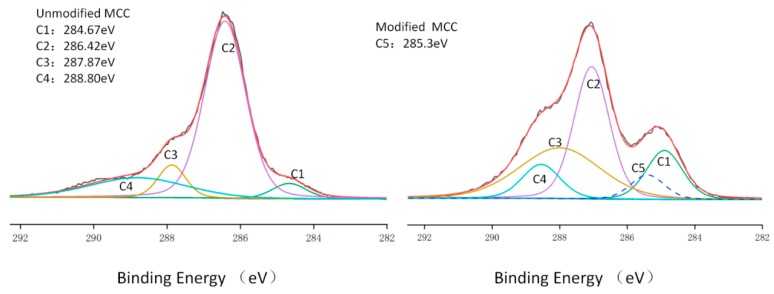
XPS high-resolution C1s spectra of MCC before and after surface modified.

Table 4 shows the percentage changes of carbon, oxygen and silicon atoms in MCC before and after PMHS modification. Due to the presence of a large number of methyl groups in PMHS, the grafting of PMHS chain onto MCC surface resulted in a decrease in O/C ratio from 79.88 to 76.62%. The PMHS grafted onto the MCC surface resulted in this change in atom amounts. After PMHS modification, the silicon content on the MCC surface significantly increased from 0 to 6.99%, the oxygen content decreased from 44.23 to 40.35%, and the carbon content decreased from 55.37 to 52.66%. After reaction, the sample was washed three times with n-hexane, the purpose was to remove the unreacted PMHS on the MCC surface because that the PMHS is soluble in n-hexane, so there will be no unreacted PMHS to bring a new Si peak. To quantify the functional groups on the surface of MCC, the high-resolution C1s spectrum was decomposed into functional groups of different carbon components (Figure 8). The unmodified MCC showed four characteristic functional groups (C1, C2, C3 and C4) which are commonly observed on the surfaces of MCC, while the PMHS–treated MCC showed evidence of surface modification, one new peak (C5 bond) appeared at 285.4 eV, respectively, in addition to the four common peaks. The C5 signal corresponded to the carbons from C–Si bonds, which was ascribed to the PMHS.

## 4. Conclusions

In summary, we developed a simple method for hydrophobic modification of MCC by using response surface methodology. The optimal reaction conditions were determined as follows: he concentration of PMHS was 0.0275%, the reaction time was 20 min, the drying temperature was 70 °C. Under the optimized reaction conditions, the contact angle of modified MCC was 141.5°. The value of Lack of Fit was not significant, which indicates that the response surface method is effective for the optimization of MCC hydrophobicity, and the prediction guidance was strong. XPS analysis results revealed that the PMHS was successfully grafted to the surface of MCC. The WCA of the MCC samples increased remarkably after PMHS modification, indicating that the surface modification of MCC by PMHS was feasible and the method was simple and efficient. 

## Figures and Tables

**Table 1:** Experimental factors and coding level.

Factors	Code and Level		
	−1	0	1
A: concentration of PMHS/(%/1000)	5	27.5	50
B: reaction time/min	10	20	30
C: drying temperature/°C	40	70	100

**Table 2:** Design-expert experimental Design table and results.

Experiment Number	A	B	C	Experimental WCA (°)	Predicted WCA (°)
1	0	0	0	140.56	140.23
2	1	0	1	138.54	138.36
3	0	1	−1	134.14	134.63
4	−1	−1	0	136.10	136.09
5	0	0	0	140.03	140.23
6	0	0	0	138.71	140.23
7	1	−1	0	136.69	137.05
8	0	−1	1	138.23	138.37
9	1	0	−1	135.86	135.68
10	0	−1	−1	136.64	136.15
11	0	0	0	141.48	140.23
12	−1	0	−1	136.08	136.26
13	1	1	0	137.89	137.90
14	−1	0	1	130.95	131.13
15	0	0	0	140.36	140.23
16	0	1	1	136.99	136.85
17	−1	1	0	132.56	132.20

**Table 3:** Analysis of experimental variance results.

Source	Sum of Squares	df	Mean Square	F Value	*p*-Value Prob > F
Model	123.12	9	13.68	19.35	0.0004
A	22.09	1	22.09	31.26	0.0008
B	4.63	1	4.63	6.56	0.0375
C	4.95	1	4.95	7.00	0.0331
AB	5.62	1	5.62	7.95	0.0258
AC	15.23	1	15.23	21.55	0.0024
A^2^	32.57	1	32.57	46.08	0.0003
B^2^	11.28	1	11.28	15.95	0.0052
C^2^	18.40	1	18.40	26.03	0.0014
A^2^C	5.95	1	5.95	8.42	0.0229
Residual	4.95	7	0.71		
Lack of Fit	0.90	3	0.30	0.30	0.8269
Pure Error	4.05	4	1.01		
Cor Total	128.07	16			

**Table 4:** The surface elements and functional groups distribution of MCC determined by XPS.

Samples	Element Concentration (%)			Atomic Ratio	Ratios of Functional Groups (C1s) (%)				
	C	O	Si	O/C	C1 (C–C/C–H)	C2 (C–O)	C3 (C=O/O–C–O)	C4 (O–C=O)	C5 (C–Si)
MCC	55.37	44.23	0	0.80	4.96	68.50	8.52	18.02	-
Modified MCC	52.66	40.35	6.99	0.77	13.97	36.85	32.52	10.12	6.53
